# A Role for Macroalgae and Cephalopods in Sustainable Eating

**DOI:** 10.3389/fpsyg.2020.01402

**Published:** 2020-07-07

**Authors:** Ole G. Mouritsen, Charlotte Vinther Schmidt

**Affiliations:** Department of Food Science, Taste for Life & Design and Consumer Behavior, University of Copenhagen, Frederiksberg, Denmark

**Keywords:** food, sustainable eating, seaweeds, squid, octopus, cuttlefish, umami

Since the sea is infinite and of unmeasured depth, many things are hidden …

*Oppian of Anazarbus: Halieutica*

*(second century Greco-Roman poet)*

It is well-established that our close ancestors, *Homo erectus*, did not evolve on the dry warm grasslands in Africa, but in coastal regions near the ocean or at great lakes (Crawford and Marsh, 1989; Cunnane et al., 2014). Apart from archaeological testimony, the most crucial argument for this statement is that only with access to plenty of marine food supplies would our ancestors be able to acquire sufficient amounts of those essential fatty acids, the super-unsaturated omega-3 and omega-6 fatty acids, in addition to certain micronutrients, like iodine, iron, copper, zinc, and selenium, which are absolutely critical for building a complex neural system and a brain with the very large brain/body weight ratio (2.1%) that is characteristic for humans (Cunnane et al., 2014; Cornish et al., 2017).

This evolutionary path is important to keep in mind when evaluating which routes to take toward a more sustainable eating behavior that is also healthy in the long run. A sustainable diet has been defined as a diet produced with little environmental impact, and which is protective and respectful of biodiversity and ecosystems, nutritionally adequate, safe, healthy, culturally acceptable, as well as economically affordable (Pimentel and Pimentel, 2003; Aleksandrowicz et al., 2016; Chai et al., 2019). The essential nutrients of marine food and the associated flavors must therefore also be taken into account when proposing a new, sustainable, healthy, and palatable path of eating behavior. According to Sproesser et al. (2019), modern diets are dominated by a high consumption of energy-dense foods, diet drinks and foods, refined foods, animal foods, oils and fats, as well as too much salt, whereas former traditional diets were characterized by consumption of basic foods (i.e., “everyday” foods), plant-based foods, grains, fruit, vegetables, and fiber. An obvious solution to eat more sustainably would be reverting to a traditional diet and include marine foods. However, the general trend of eating behaviors, in particular in the Western world and affluent countries, is that the diet contains less marine food sources than before, and more meat and highly processed plant-based foods and oils (Willett et al., 2019, and references therein).

A little background is in order to appreciate what is at stake: It is universally found in all species with neural systems that the neural membranes, including the brain, contain about 60% fat (dry weight) of which more than half are super-unsaturated fatty acids (Cunnane et al., 2014), in particular omega-6 arachidonic acid (20:4) and adrenic acid (22:4), and omega-3 docosahexaenoic acid (DHA, 22:6) and eicosapentaenoic acid (EPA, 22:5). Even more striking is that the total amount of all omega-6 fatty acids in the brain is almost the same as the total amount of all omega-3 fatty acids, rendering the ratio omega-6/omega-3 in the brain close to 1 (Crawford, 2007). All these fatty acids are essential fatty acids, i.e., our bodies have only insufficient mechanisms to synthesize them and hence they have to be acquired via our food. The access to these fatty acids is therefore considered a determining factor in the evolution of modern humans (Crawford and Marsh, 1989).

Comparison of the diet of earlier populations with present populations reveals many differences, e.g., the content of dietary fibers (Makki et al., 2018), but maybe the most striking difference is the omega-6/omega-3 ratio that now appears to be way out of balance and typically ranging from 5 to 25 and increasing (Simopoulos, 2002; Mouritsen, 2016). This gross imbalance has been suggested to be a main reason for the skyrocketing of neural related diseases, such as depression, bipolar disorders, and a wide range of other mental diseases that is now becoming a major and very costly global burden of human ill health (GBD 2017 Diet Collaborators, 2019). Researchers have raised concerns about this development not least in young subjects, suggesting that the human brain is under siege (Cunnane et al., 2014).

There are several reasons for the raise in the omega-6/omega-3 ratio of the diet (Simopoulos, 2002; Crawford, 2007). One is the growth in production of cheap omega-6-containing plant oils during the second half of the 20th century; another is the stagnation in fisheries and limitations in the growth of aquaculture due to environmental considerations. The requirement for omega-3 fats in the diet poses constraints on the calculations underlying the proposal for a sustainable, healthy, and nutritious diet for a growing global population as outlined in the EAT-Lancet Commission report (Willett et al., 2019).

So where do the super-unsaturated fatty acids come from? They are all synthesized at the bottom of the food web by the algae, both the microalgae and the macroalgae (seaweeds). Only these organisms have the enzyme systems required to produce the super-unsaturated fatty acids from other fatty acids (Cornish et al., 2015). Neither we, other animals, nor plants can do that. The super-unsaturated fatty acids ascend through the food web and become accumulated particularly in marine organisms, such as fish, shellfish, and some mammals. This is where humans would usually source their essential fatty acids like DHA and EPA. However, in the context of sustainable eating behavior, eating from the top of the food web may not be the wisest way to use the global food resources, since typically 90% of the nutrients are lost when the food goes through each trophic level. Eating more sustainably points to eating closer to the bottom of the food web, in particular from the sea (Costello et al., 2019). Eating more sustainable marine food resources also implies considering eating marine species that are currently not exploited, not conventionally considered as food, or simply are only little known by consumers. We shall in the following address these routes to sustainable eating with main focus on macroalgae and mollusks.

Algae constitute a very heterogeneous diverse group of unicellular (microalgae like phytoplankton and cyanobacteria) and multicellular (macroalgae like marine seaweeds, cf. Figure 1) organisms. Being photosynthetic, the algae are responsible for producing most of the atmosphere's oxygen and fixating the most carbon dioxide (Chapman, 2013). They are found in all climatic belts on the planet and they can be harvested sustainable in the wild or farmed in aquaculture (Mouritsen, 2013; Pérez-Lloréns et al., 2018). Most seaweeds are considered edible and tasty (Mouritsen, 2017; Mouritsen et al., 2019a) and some with umami flavor (Mouritsen et al., 2019b). Seaweeds are an important part of the diet in many Eastern countries, but only sparsely exploited and eaten in the Western world. We would like to point out that seaweeds have to be an important part of eating more green in the future.

**Figure 1:**
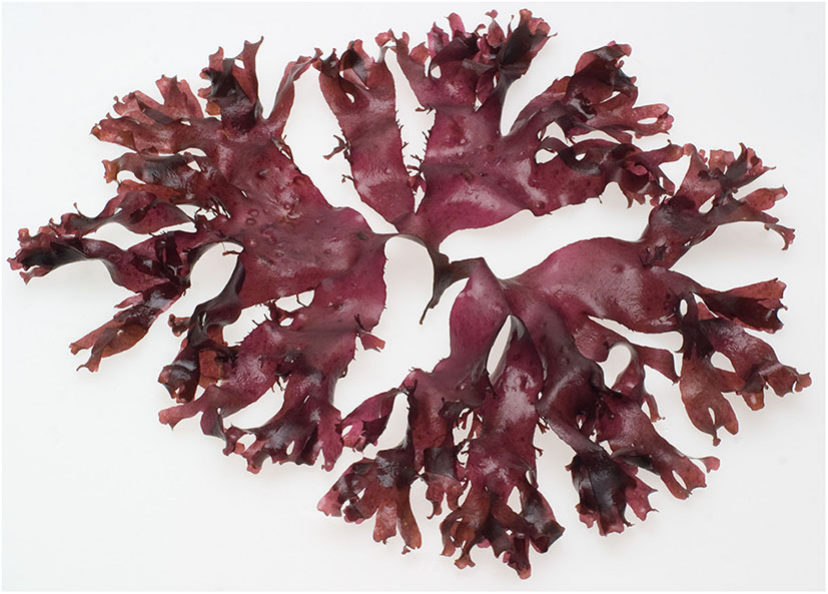
An edible seaweed, dulse (*Palmaria palmata*), a delectable seaweed with a considerable umami potential (Permission to reprint by Jonas Drotner Mouritsen).

There are about 10,000 different species of seaweeds, and about 500 are exploited as food or food ingredients. The global production, most of which is derived from aquaculture (97%), amounts to about 30 million metric tons (FAO, 2018a). The future scenario of seaweed farming involves multi-trophic cultures covering also fish and mollusks (Ashkenazi et al., 2018). Seaweeds are generally high in both macro- and micronutrients as well as vitamins, and being algae, they synthesize super-unsaturated omega-3 fatty acids, in particular EPA (Mouritsen, 2013; Shannon and Abu-Ghannam, 2019). In relation to economic sustainability, a recent calculation has shown that the economic value of marine vegetation is almost 20 times higher than that of terrestrial forests per hectare (Pérez-Lloréns et al., 2018).

Turning to marine animals, world fisheries are under severe pressure due to overfishing, dwindling and endangered wild populations, as well as severe impact from the industrial fishing methods, although measures have been taken to control and manage wild fish populations (FAO, 2018b). Aquaculture currently account for almost half of the world fisheries (FAO, 2018b), but due to problems with sustainability, pollution, discharge of excess nutrients, and pressure on the natural marine ecosystems it will be difficult to scale up further to meet world demands for fish and shellfish. At the same time, a large volume of scrap fish and bycatch, although getting more tightly regulated by international agencies, are either discarded or processed for feed to livestock and fish farms. Some of the “scrap” fish such as sprat are mostly caught commercially for feed and never directly reach the consumer before it has been turned into pork or farmed salmon, even if sprat actually has high gastronomic value. We are simply not accustomed to eating it. The latter holds also true for other fish or mollusk species, such as Pacific oysters or round goby, which traditionally may not be known in a particular food culture but may have ventured into the local waters as an invasive species adapting to climate changes.

Cephalopods (squid, cuttlefish, and octopus; cf. Figure 2) are an example of mollusks that are eaten in some parts of the world and not in others, even if they are abundant in the local waters. In contrast to the dwindling populations of finfish, it was recently reported that the global populations of all fished cephalopod species appear to be on the rise and have been so for the last almost 60 years, possibly having benefited from changes in the ocean (Doubleday et al., 2016). It immediately poses the question: why do we not eat more of them (Mouritsen and Styrbæk, 2018)? Out of the 800 global species, about 30 are used as human food, and the annual catch amounts to 5% of the world fisheries and it is increasing rapidly (FAO, 2018b). Cephalopods are thus a rather unexploited marine crop, despite being high in protein, minerals, trace elements, B_12_-vitamin, and fair amounts of super-unsaturated fatty acids (DHA and EPA) although the contents of fatty acids are much lower than in fatty fish but similar to that of lean fish.

**Figure 2:**
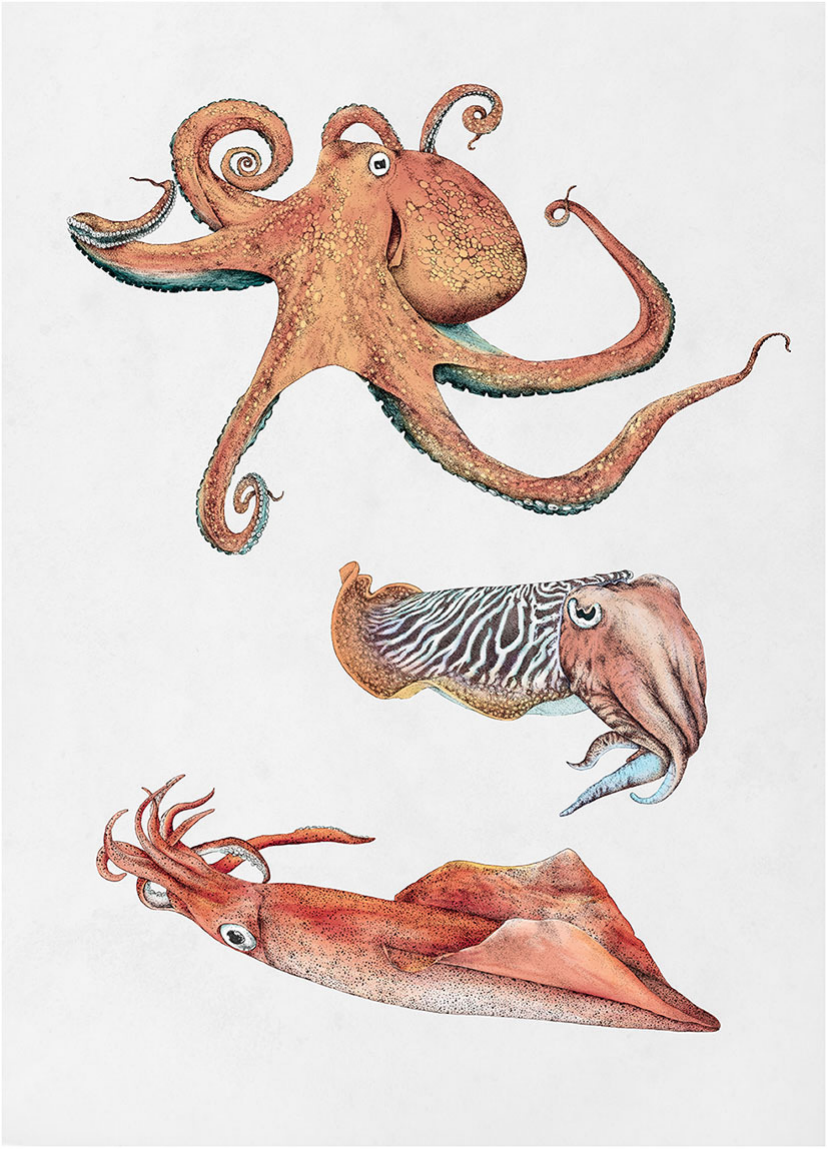
Illustrations of the three most common classes of cephalopods that are used for eating: octopus, cuttlefish, and squid (Permission to reprint by Ene Es).

So, there appears to be good reasons to consider consuming more seaweeds and cephalopods when changing eating behavior toward more sustainable and healthy eating. But what are the obstacles? Obviously, tradition, culture, and availability are key factors (Mouritsen and Styrbæk, 2018; Pérez-Lloréns et al., 2020) and often also taste and texture are limiting factors. Our opinion is that by addressing precisely these two sensory aspects of impacting eating behavior we may have a chance of stimulating changes. Interestingly enough, often marine flavor is claimed to be a barrier for eating seaweeds, despite of the fact that desirable umami taste often accompanies seaweeds, whereas undesired texture is mentioned as an obstacle for appreciating cephalopods, like squid and octopus (Mouritsen and Styrbæk, 2018). Culinary sciences, gastrophysics, and cooking practices can be invoked to confront these issues (Faxholm et al., 2018).

Umami as a basic taste was first identified in the kind of seaweed (konbu, *Saccharina japonica*) that constitutes a key component in the Japanese soup broth dashi that is associated with deliciousness (Mouritsen and Styrbæk, 2014). Dashi is an aqueous extract of konbu and it can be used to impart umami taste to other ingredients, not least green food like vegetables, which often lacks umami taste (Schmidt and Mouritsen, 2020). “Umamification” of vegetables may be one route to eating more green, either by using dashi or seaweeds as whole foods (Mouritsen, 2013, 2018). In the present authors' opinion, “umamification” is a key to meeting our craving for umami taste, shaped by more than 2 million years of the evolution of humans as meat eaters (Wrangham, 2009).

Turning toward cephalopods as a source of food, they are as much a source of protein as other marine foodstuff and they are rich in those compounds that elicit umami and sweetness (Faxholm et al., 2018). The texture can be challenging due to the abundance and nature of the collagen in cephalopod muscles, but as with other types of meat this can be handled by culinary insight, craftsmanship, and scientific knowledge (Faxholm et al., 2018; Styrbæk and Mouritsen, 2020) as is well-known in the Japanese cuisine. It should be noticed that cephalopods are meat and from animals, even if they are mollusks and invertebrates. As it is becoming clearer that some of the cephalopods, and most certainly octopus, have a very advanced neural system and a brain that may be seat of consciousness like vertebrates (Cambridge Declaration of Consciousness, 2012), some may stay away from eating them. Steps have therefore also been taken to develop methods for humane slaughtering of cephalopods (Fiorito et al., 2015; Holden-Dye et al., 2020). On the other hand, cephalopod meat may be an attractive substitute for meat from land animals for consumers that absolutely want to eat meat but has a keen eye to more sustainable eating. Here the growing and thriving global populations of cephalopods may play an important role. Since cephalopod meat has umami taste, small amounts of this meat could help make a vegetable diet more delectable for flexitarians.

In addition to sensory and physiological factors, there may be social and psychological factors preventing one from eating strange things like seaweeds and cephalopods. The words “weed” or “wrack” that are used for some seaweeds thrown ashore from the sea, lying smelling and rotting at the foreshore, do not give good connotations to food. However, this is very dependent on culture and language. In Western food cultures, attempts have been made to give a more positive image to seaweeds by proposing terms like “sea vegetables,” “sea greens,” or “ocean greens” and to further boost them by using plus words like “superfood” or “future food.” It is likely that the globalization of food, the increasing influence of Eastern food cultures, along with a focus on the health benefits of eating seaweeds are responsible for the current increase in the interest and consumption of seaweeds in both North America and in Europe (Organic Monitor, 2014; Chapman et al., 2015; Lucas et al., 2019; Pérez-Lloréns, 2019).

Finally, gender differences may play a role in the further move toward more sustainable eating involving seaweeds and cephalopods. Women have showed themselves as an avantgarde regarding adopting an environmentally more sustainable consumer behavior (Iris et al., 2018) including a more green diet (O'Doherty Jensen and Holm, 1999) which also incorporates seaweeds and nutritional microalgae like Spirulina and *Chlorella*. In this respect males are lacking behind. However, here cephalopods may come in as a rescue when used as a condiment and flavoring agent for, e.g., vegetables, conforming to the observation that males tend to consider meat as a masculine form for food (Ruby and Heine, 2011; Graça et al., 2015). In any case, more knowledge and dissemination of scientific knowledge coupled with gastronomic innovation will be important for promoting and substantiating a move toward sustainable eating involving macroalgae and cephalopods (Sørensen and Mouritsen, 2019).

## Author Contributions

OM conceived the work and wrote the first draft. CV did further literature research for the paper. Both authors completed the manuscript and provided approval for publication of this work.

## Conflict of Interest

The authors declare that the research was conducted in the absence of any commercial or financial relationships that could be construed as a potential conflict of interest.
